# Maximizing Biomass Production and Carotenoid-like Pigments Yield in *Kocuria sediminis* As04 Through Culture Optimization

**DOI:** 10.3390/microorganisms13071555

**Published:** 2025-07-02

**Authors:** Daniela Jakeline López-Mora, Andrea Goreti Flores-Dávalos, Miguel Angel Lorenzo-Santiago, Beatriz Genoveva Guardado-Fierros, Jacobo Rodriguez-Campos, Silvia Maribel Contreras-Ramos

**Affiliations:** 1 Unidad de Tecnología Ambiental, Centro de Investigación y Asistencia en Tecnología y Diseño del Estado de Jalisco A.C. (CIATEJ), Normalistas No. 800, Colinas de la Normal, Guadalajara C.P. 44270, Jalisco, Mexico; djmora51@gmail.com (D.J.L.-M.); floresgoreti01@gmail.com (A.G.F.-D.); beguardado_al@ciatej.edu.mx (B.G.G.-F.); 2 Unidad de Servicios Analíticos y Metrológicos, Centro de Investigación y Asistencia en Tecnología y Diseño del Estado de Jalisco A.C. (CIATEJ), Guadalajara C.P. 44270, Jalisco, Mexico; jarodriguez@ciatej.mx

**Keywords:** *Kocuria sediminis*, bacterial biomass, carotenoids, Taguchi design, secondary metabolites

## Abstract

The global chemical pigment industry faces environmental challenges despite its economic importance. This study investigates the potential of *Kocuria sediminis* AS04, an airborne isolate, for sustainable pigment and biomass production. Microbial kinetics were evaluated under Taguchi design conditions with temperature (30, 34, and 38 °C), stirring speed (110, 120, and 130), and pH (6.0, 6.5, and 7.0), measuring biomass through dry weight and viable cells, pigment production, and identification of its pigment using UPLC-MS/MS; structural and chemical characterization of biomass was conducted using SEM and FTIR. Among the tested conditions, the treatment at 30 °C, 130 rpm, and pH 6.5 resulted in the highest CFU count (5.7 × 10^9^ CFU mL^−1^) and the greatest biomass yield (13.3 g L^−1^). In contrast, the highest pigment yield (0.0016 mg g^−1^) was obtained at 38 °C, 130 rpm, and pH 6.0. Cell extracts identified key carotenoid compounds such as β-cryptoxanthin, Rhodovibrin, and other precursors. These findings highlight the potential of *Kocuria sediminis* AS04 as a sustainable source of pigments and valuable bioproducts, offering promising alternatives for eco-friendly industrial applications.

## 1. Introduction

Some genera identified as PGP bacteria (PGPB) are *Bacillus*, *Pseudomonas*, *Kocuria*, *Azospirillum*, *Arthrobacter*, *Azotobacter*, *Enterobacter*, *Klebsiella*, *Paraburkholderia*, *Serratia*, *Sphingomonas*, *Rhizobium*, etc. [1,2]. These bacteria are also potential candidates for bioremediation of pesticide-contaminated agricultural fields [1] and heavy metal remediation [3].

*Kocuria* belongs to the phylum *Actinobacteria*, class *Actinobacteria*, order Micrococcales, and family Micrococcaceae. Its cells are coccoid-shaped, Gram-positive, aerobic, catalase-positive, non-motile, and non-spore-forming. Most *Kocuria* strains grow between 20 and 37 °C, with some exceptions that are outside this range [4]. This genus can tolerate pH ranging from 6.0 to 10.0 and concentrations of up to 5.0% NaCl. On tryptic soy agar (TSA), *Kocuria* colonies typically appear pinkish-orange, irregular in shape, with entire margins, an opaque texture, and a diameter of 1–2 µm [5]. The main sources of *Kocuria* isolation are rhizospheric soil [6], marine environments [7], sediments [5], and roots [8]. Recently, some *Kocuria* spp. were isolated from the air and showed the capacity to promote plant growth (PGP) [9].

Some bacteria classified as PGPB can produce pigments such as riboflavin, lycopene, β-carotene, canthaxanthin, indigoidine, prodigiosin, pyocyanin, violacein, zeaxanthin, xhantomonadin, etc. [10]. These pigments have many applications in the pharmaceutical, food, cosmetics, and textile industries. Bacterial pigments have been extracted from *Flavobacterium* sp., *A. aurantiacum*, *Micrococcus* sp., *P. aeruginosa*, *S. marcescens*, *Chromobacterium* spp., and *Rheinheimera* spp. [11]. New technologies have improved the extraction and commercialization of bacterial pigments through innovations in genetic engineering, fermentation, and downstream processing [12,13]. Techniques such as CRISPR-Cas9 and metabolic pathway optimization allow bacteria to be modified for higher pigment yields by overexpressing biosynthetic genes or redirecting metabolic flux [14,15].

Bacterial pigments, as a natural alternative for different industrial applications, have gained great relevance since synthetic pigments have caused considerable environmental pollution, adverse toxicological side effects, hyperallergenic nature, and carcinogenicity [16]. This alternative attracts the industry’s attention due to its non-toxic, easily degradable, and eco-friendly nature. The high production cost compared to synthetic analogs remains challenging for its marketability and economic viability [10]. However, the market for natural pigments is fast growing due to the globalization of research trends and customer inclinations towards a healthier lifestyle [16].

Carotenoids are natural, fat-soluble colorants with nutritional and antioxidant properties and can be classified into two main groups: carotenes and xanthophylls. The first group is hydrocarbons, such as lycopene and β-carotene, whereas the second group incorporates oxygenated functions, such as hydroxyl, methoxy, carboxyl, keto, or epoxy groups, including lutein, β-cryptoxanthin, zeaxanthin, and fucoxanthin [17]. These compounds, responsible for the characteristic yellow, orange, and red pigmentation, comprise over 700 known structures, with more than 95% sharing a 40-carbon backbone [18]. The global carotenoids market is projected to reach USD 1.85 billion by the end of 2026, and their prices can vary depending on the type of carotenoid; for example, they can range from 300 to 3000 USD/kg of β-carotene or from 2500 to 10,000 USD/kg of astaxanthin [19]. Although synthetic production remains prevalent due to lower costs, microbial production offers a promising alternative; for example, while synthetic astaxanthin is estimated at 1000 USD/kg, microbial production using *Haematococcus pluvialis* may reduce costs to around 552 USD/kg [20].

Optimization of microbial cultivation can help to achieve a greater yield in pigment production. Temperature, pH, light, aeration, and agitation can influence carotenoid production [20]. Optimizing such factors can lead to the possibility that carotenoids could be competitive in the market shortly. Brahma and Duta [21] and Mendes-Silva et al. [18] found that optimizing factors such as light exposure, temperature at 30 °C, aeration for oxygen transfer, and greater agitation (250 rpm) increases carotenoid production for *Kocuria palustris*. Some authors have reported that *Kocuria marina* DAGII grows at 25 °C with a rotation speed of 150 rpm, *Kocuria* spp. DAB-1Y shows optimal growth at 30 °C and 120 rpm, while *Kocuria flava* grows best at 37 °C with shaking at 180 rpm [21,22,23]. However, these parameters may vary depending on the desired product of the fermentation process.

A few pigments produced by the *Kocuria* genus have been identified, including cryptoxanthin [21] and sarcinaxanthin [18]. A literature review reveals that most studies on *Kocuria sediminis* are limited to isolation, taxonomic identification, environmental isolation, and degradation of compounds [5,24,25]. Few works have maximized the production [21].

Microbial carotenoids are of interest due to their antioxidant, coloring, and photoprotective properties, which make them attractive to the food, cosmetic, and pharmaceutical industries [26,27]. Although *Kocuria* species, such as *Kocuria rosea*, have been reported to produce carotenoid pigments [28], little research has been conducted on *Kocuria sediminis*. This species has received limited attention despite being taxonomically described over a decade ago [5]. This study selected it for investigation due to its origin as a bioaerosol isolate, suggesting a possible adaptation to oxidative stress, a condition often associated with carotenoid production as a protective mechanism [29,30]. Additionally, the distinctive pigmentation observed during preliminary culturing motivated this study to systematically evaluate its biomass and pigment production under controlled conditions. Thus, this study addresses the knowledge gap regarding *Kocuria sediminis* and factors such as pH, temperature, and stirring speed that maximize biomass production and carotenoid-like pigment yield.

## 2. Materials and Methods

### 2.1. Isolation of Kocuria Strain from Airborne Samples

Isolation, identification, and PGPB characterization of the *Kocuria* strain As04 used in this work were previously described by Guardado Fierros et al. [31]. Briefly, air samples of 100 L each were collected from the metropolitan area of Guadalajara, Mexico, “San Juan de Dios” (20° 40′31″ N; 103° 20′24″ W). Sampling was conducted using an air sampler gun (Millipore^®^ M air T, Billerica, MA, USA), and the samples were spread on plates containing Luria-Bertani agar (LB). The most representative morphotype strains were isolated in LB medium and biotyped by the MALDI-TOF system (Bruker Daltonics, Leipzig, Germany) described by Tuesta-Popolizio et al. [32].

Molecular identification was obtained through matrix-assisted laser desorption/ionization coupled to mass spectrometry-time-of-flight (MALDI-TOF-MS) using a MICROFLEX LT mass spectrometer (Bruker Daltonics, Bremen, Germany) with single colony biomass (DT Method, Bruker Daltonics) [9]. This technique adheres to the manual’s MALDI Biotarget 96 instructions, with some modifications [33]. Taxonomic identification was performed by comparing the mass spectra with the BDAL database using MALDI-Biotyper 3.4 software (Bruker Daltonics, Billerica, MA, USA). The spectrometer was calibrated using the Bacterial Protein Test Standard (Bruker Daltonics). Only isolates with a score > 1.700, meeting the identification criteria at the genus level, were considered positive.

Molecular identification was performed using FastDNA Spin Kit^®^ (MP Biomedicals, Solon, OH, USA) for soil and was utilized for DNA extraction from representative genera determined by MALDI-TOF MS. DNA concentration was assessed using UV-VIS spectrophotometry (NanoDrop-2000, Thermo Scientific, Waltham, MA, USA) and stored at −20 °C until use [9]. Sequencing was performed at Sanger PSOMAGEN INC. (Rockville, MD, USA) using primers 27F and 1492R [9]. The final sequences were compared against the NCBI 16S database (BLAST, Rockville Pike, MD, USA) and deposited in the GenBank database (http://www.ncbi.nlm.nih.gov/BLAST, accessed on 11 April 2025). The *Kocuria* strain morphology was positive-cocci in Gram stain, with orange-red pigmentation and a 2.130 score by MALDI-TOF, which guaranteed the *Kocuria* genus. Molecular identification was carried out through 16S rRNA gene sequencing, yielding a fragment of 532 base pairs. The sequence showed 100% query coverage and 98.31% identity compared to reference sequences in the GenBank database, indicating high similarity to known *Kocuria sediminis* strains. The corresponding GenBank accession number for the closest match is OP934048.

### 2.2. Culture Optimization

#### Taguchi Experimental Design

The Taguchi design was chosen for its efficiency in reducing the number of experiments while effectively identifying significant factors, making it suitable for preliminary optimization compared to more complex methods. The experimental design was based on a Taguchi L9 orthogonal array (nine combinations) and was generated using Minitab 20.3 software. The experiment was established with three different variables at three levels to optimize biomass and pigment production: temperature (30, 34, and 38 °C) and orbital shaking (110, 120, and 130 rpm) using an incubator with orbital shaking INO 650V-7- (SEV-PRENDO, Puebla, México); and pH (6.0, 6.5 and 7.0) using a potentiometer Hanna Instruments HI 3512 Bench Meter (Hanna Instruments, Smithfield, RI, USA), according to Table 1. All conditions were evaluated in triplicate. The selected conditions were based on the original isolation parameters of *Kocuria sediminis* AS04 (airborne), which included a temperature of 30 °C, pH 7.0, and agitation at 100 rpm. Before conducting kinetic studies, the strain was tested under various pH levels (up to 9), temperatures (up to 40 °C), and agitation speeds (up to 150 rpm) (unpublished data). However, growth was limited under extreme conditions. The final parameters were chosen for their consistent growth.

### 2.3. Microbial Kinetics from Kocuria sediminis As04

#### 2.3.1. Pre-Inoculum Production

The *Kocuria sediminis* As04, previously isolated from an airborne environment and described by Guardado-Fierros et al. [9], was reseeded in Tryptone Soy Agar (TSA) (Difco, MI, USA) and incubated for 48 h at 30 °C. Afterward, three flasks were prepared with 400 mL of Tryptone Soy Broth (TSB) (Difco, MI, USA) at pH 6.9 and were sterilized at 121 °C for 20 min at 15 psi in a BKQ-B75II autoclave (Biobase, Jinan, Shandong, China). Subsequently, the TSB was inoculated with 10% of the strain and incubated at 30 °C ± 2 and 100 rpm for 24 h (pre-inoculum conditions).

At 48 h, 5 mL samples from each flask were collected for viable count and optical density (OD). The number of CFUs from the pre-inoculum was determined using Plate Count Agar (PCA). The OD was measured at 600 nm using a DR 5000TM UV–Vis spectrophotometer (Hach, Mississauga, ON, Canada). For all analyses, uninoculated TSB was used as a negative control.

#### 2.3.2. Taguchi Experiment

Three flasks with 1.5 L of TSB were sterilized at 121 °C for 20 min at 15 psi, each adjusted to pH 6.0, 6.5, and 7.0 using sodium hydroxide at 0.5 N and citric acid at 1 N. Equation (1) calculated the volume of pre-inoculum and TSB necessary to start with an OD of 0.1. All treatments were conducted in triplicate.

C1V1 = C2V2(1)

C1 is the initial concentration, V1 is the initial volume, C2 is the final concentration, and V2 is the final volume. All Taguchi treatments were established in 150 mL sterile flasks and inoculated with pre-inoculated culture at the corresponding pH. The nine treatments were separated into three stages based on the agitation utilized (110, 120, and 130 rpm). All flasks were incubated under the temperature conditions and stirring speeds according to Table 1. A sample of 20 mL of culture was used to determine OD, CFU, and biomass as dry weight at 0, 4, 8, 12, 24, and 48 h. Total carotenoid pigments were determined at the end of fermentation in each treatment, and biomass was separated by pigment extraction from all Taguchi treatments. The treatments with higher biomass were reserved for subsequent chromatographic (MS/MS) analysis for pigment identification. In addition, a sample of culture (25 mL) was used for structural and chemical characterization by SEM and FTIR of the biomass.

#### 2.3.3. Determination of Biomass and Total Viable Count

A 5 mL sample taken at 0, 4, 8, 12, 24, and 48 h was used to measure OD, and 10 mL was used for dry weight. Dry weight was analyzed using the method described by ASTM D2974-20, with some modifications [34]. Filter paper with a pore size of 2 µm was cut into 10 cm^2^ squares and dried at 40 °C ± 2 for 12 h to determine biomass as dry weight. The filter was cooled in a desiccator at 25 °C with a relative humidity between 5 and 10% before weighing to prevent moisture uptake from ambient air. The paper squares were placed on the funnel, and the 10 mL samples were filtered. The biomass retained on the paper was dried for 24 h at 40 °C ± 2. Once the time elapsed, the filter paper was weighed, and its percentage of dry weight was estimated with Equation (2). The dry weight was calculated:

(2) $$Dry\;weight=\left(Wdry-Wcontainer\right)\times 100$$

After drying, the container’s combined weight (g) and the dried sample were recorded as W_dry_, while the weight (g) of the empty container or filter paper was denoted as W_container_. The difference between these values represents the dry biomass obtained from the 10 mL sample. The value was multiplied by 100 to express the result as grams per liter (g L^−1^). This calculation reflects the sample’s dry weight or total solids content. Simultaneously, 1 mL of the sample was analyzed for the total viable count. Serial dilutions were prepared in 2 mL Eppendorf tubes by adding 0.9 mL of sterile saline solution (0.9% *w*/*v* NaCl) to 0.1 mL of the sample. Petri plates were prepared using standard agar methods, and 100 µL of each dilution was spread on the plates. The plates were incubated at 30 °C ± 2 for 24 h, after which the colonies were counted to determine the CFU mL^−1^ in each experiment.

The specific growth rate (*μmax*) and doubling time (DT) were calculated using Equations (3) and (4), which are based on exponential growth kinetics.

(3) $$\mu max=\frac{lnX2-lnX1}{t2-t1}$$

(4) $$DT=\frac{ln2}{\mu max}$$

where *X*_1_ and *X*_2_ are biomass concentrations at times *t*_1_ and *t*_2_ during the exponential phase, DT is the time to double the biomass, and *μmax* is expressed in h^−1^.

#### 2.3.4. Determination of Total Carotenoid Pigments

For total pigment determination, 2 mL of bacterial culture was centrifuged in a 15 mL conical tube. After centrifugation, the supernatant was removed, and 2 mL of an acetone–methanol solution (7:2 *v*/*v*) was added to the biomass. The samples were stored in the dark at 6 °C with slow agitation (15 rpm) until the cells lost color. The samples were centrifuged at 4000 rpm at 2 °C ± 2, and the supernatant was subsequently filtered using a 0.20 µm filter. The absorbance of the sample was measured at 473 nm using a DR 5000™ UV–Vis spectrophotometer (Hach, Mississauga, ON, Canada). Pigment concentrations were calculated using Equation (5), according to Zhi et al. [35]. The total pigments produced by *Kocuria sediminis* As04 were quantified using lycopene (Sigma Aldrich, Saint Louis, MO, USA, ≥98%) as a reference standard, with a molar extinction coefficient (ε) of 1.72 × 10^5^ L·mol^−1^·cm^−1^, according to Fish et al. [36]. Uninoculated TSB was used as a negative control. The total carotenoids were calculated in mg g^−1^.

(5) $$Total\;carotenoids=\frac{A\times V\times F}{\varepsilon \times L\times DW}$$

where *A* is the absorbance of the extract for carotenoids; *V* is the total volume of the sample (mL); F is the sample dilution factor; *ε* is the molar extinction coefficient of the lycopene in the solvent used (L·mol^−1^·cm^−1^); L represents the path length (cm); and *DW* is the initial amount (g) of dry cells.

### 2.4. Pigment Identification by MS/MS

#### 2.4.1. Pigment Extraction

Intracellular bacterial pigments were extracted from 25 mL of culture at the end of fermentation in the treatment where higher biomass was produced. The culture was centrifuged at 4000 rpm and 4 °C ± 2 for 10 min. The resulting cell pellet, enriched with pigments, was subjected to further processing. In a 15 mL conical tube, shielded from light with aluminum foil and immersed in an ice bath, 400 µL of water and 400 µL of methanol were sequentially introduced, followed by one hour of gentle agitation at 15 rpm. Subsequently, 800 µL of chloroform was added, and the pellet was further agitated until complete dissolution. The sample was allowed to stand at room temperature for one hour to promote phase separation. The pigmented solution was transferred to a 2 mL microcentrifuge tube protected from light with aluminum foil. This solution was centrifuged at 13,000 rpm for 10 min to separate the phases. The lower chloroform layer containing the extracted pigments was then carefully transferred to a new 2 mL microcentrifuge tube and filtered through a 0.2 µm nylon membrane to remove residual particulates.

The desiccation of the filtered extract was carried out using a Vacuum Concentrator DNA 130 (Thermo Scientific, Holbrook, NY, USA) at 35 °C ± 2 for 45 min, and the samples were stored at −20 °C ± 2 until their analysis. For chromatographic analysis, the samples were dissolved in 500 µL of mass-grade methanol [37], and as a negative control, uninoculated was used TSB.

#### 2.4.2. Chromatographic and Mass Spectrometry Conditions and Data Processing

The pigmented extracts obtained from *Kocuria sediminis* As04 were injected into the ultra-performance liquid chromatography–time-of-flight mass spectrometry (UPLC-Q-TOF/MS Xevo) (G2-XS model, Waters, Milford, MA, USA). The sample volume was 5 μL each time, and the liquid flow rate was 0.45 mL min^−1^. The mobile phase comprised a 0.1% formic acid aqueous solution (mobile phase A) and a 0.1% formic acid acetonitrile solution (mobile phase B). The gradient elution procedure started with 5% of B, increasing to 95% in 10 min; it was maintained for 5 min and then returned to the initial condition. All separations were carried out utilizing a Waters ACQUITY UHPLC^®^ HSS T3 (Waters, Milford, MA, USA, 2.1 mm × 100 mm, 1.8 μm particle size) at a column temperature of 40 °C and an autosampler temperature of 7 °C.

The MS^E^ acquisition mode was set to positive polarity. The high collision energy ranged from 20 to 55 eV, whereas the low collision energy was fixed at 6 eV, and the ionization mode was set as electron spray ionization (ESI). Scan spectra ranged from 50 to 700 *m*/*z*, with a scan velocity of 0.10 s. Ionization parameters were as follows: the cone voltage was 15 V, and the capillary voltage was 2.5 kV in the negative mode. The desolvation temperature was fixed at 550 °C, and the ion source temperature remained at 120 °C. Desolvation gas (N_2_) flowed at 1000 L h^−1^, while the cone gas (N_2_) flowed at 50 L h^−1^.

Data were acquired using MassLynx software (V4.1. Waters Corporation, Milford, MA, USA). During data acquisition, 200 pg mL^−1^ of Leucin enkephalin (Leu-enkephalin) solution (Waters, USA) was infused continuously at 5 µL min^−1^ via a lock spray interface to monitor the mass-to-charge ratio (*m*/*z*). Leu-enkephalin ions were generated in negative mode at *m*/*z* 554.2615 [M-H] to ensure mass accuracy and reproducibility.

The UNIFI 1.8.0 platform (Waters, Manchester, UK) was employed to analyze the pigment molecules in the samples. The raw data were processed using MassLynx software, which included correction and acquisition. First, they were imported directly into the UNIFI library for processing. Tentative identification of main compounds was performed with the following parameters: retention time (RT) range of 0–20 min, tolerance for retention time: 0.1 min, target match tolerance ±10 ppm, high energy intensity threshold 500 counts, low energy intensity threshold 1000 counts to reduce background noise, and adduct form selected as H- for negative ionization mode.

An automated matching analysis was conducted on the UNIFI platform, utilizing precise mass and fragment ions. Pigments were identified by combining accurate masses, *MS/MS* fragment cleavage patterns, UNIFI platform matching results, and literature references. The mass spectra of each compound were manually checked to verify that the molecular fragments predicted by the software were from a single compound. The unique mass spectrometry fragment patterns observed were compared with the fragment ions documented in the literature and data libraries such as PubChem (https://pubchem.ncbi.nlm.nih.gov/, accessed on 10 March 2025), Massbank (https://massbank.eu/MassBank/, accessed on 10 March 2025), and ChemSpider (https://www.chemspider.com, accessed on 11 March 2025) for the final identification of pigmented compounds [38,39,40].

### 2.5. Structural and Chemical Characterization by SEM and FTIR

To determine the structure and the functional groups in the bacterium *Kocuria sediminis* As04, 25 mL of the best treatment (Taguchi experiment) was centrifuged at 4000 rpm at 4 °C ± 2 for 10 min. The precipitate was added to 2 mL vials and filled with 1.5 mL of ethanol. The vials were centrifuged at 4000 rpm for 5 min (the process was repeated three times). The extract was dried using a Vacuum Concentrator DNA 130 (Thermo Scientific, Holbrook, NY, USA) for 45 min at 35 °C ± 2 and stored until analysis.

Functional group analysis was performed using a Fourier transform infrared (FTIR) spectrometer, Cary 630 (Agilent Technologies, Santa Clara, CA, USA). The infrared region was 450−4000 cm^−1^ in absorbance mode, with a resolution of 4 cm^−1^ and 10 scans. The micrographs were taken using scanning electron microscopy (SEM), JEOL model JSM−6010LA (Jeol, Tokyo, Japan). The samples were prepared in a vacuum, coated with gold to avoid static load, and operated at 15 kV, with magnifications of 1000× and 5000× to observe the morphology.

### 2.6. Statistical Analysis

All results were obtained using variance analyses (ANOVA) and the Tukey test with a probability level of 5% (*p* < 0.05) with a confidence level of 95% to analyze whether there is a significant difference in biomass and pigment production with each treatment applied. Surface response graphs were generated (Minitab 20.3, State College, PA, USA), and Tukey Studentized Rank Analysis (HSD) Type III was performed (XLSTAT 2020.2.1, Denver, CO, USA).

## 3. Results and Discussion

### 3.1. Isolation and Identification

**Figure 1:**
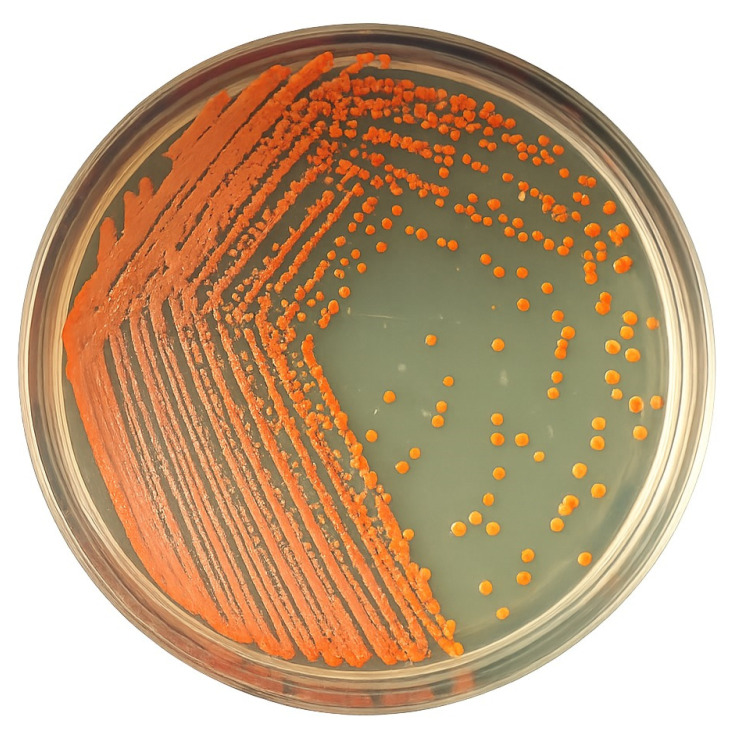
*Kocuria sediminis* AS04 culture showing the biomass with orange-red pigmentation.

The bacteria isolated from the San Juan site, identified with the code As04, exhibited morphological characteristics consistent with bacteria of the genus *Kocuria*. According to the Gram stain, the organism is a Gram-positive coccus, which aligns with the taxonomy of the genus belonging to the *Micrococcaceae* family. Additionally, the colony exhibited orange-red pigmentation (Figure 1), a typical trait of *Kocuria* species that can produce carotenoid pigments [41,42]. *Kocuria sediminis* is commonly found in sedimentary environments. Its isolation suggests this bacterium’s possible resilience and adaptability to other matrices, which could have potential implications for environmental or biotechnological applications [43].

### 3.2. Kocuria sediminis Culture Optimization

According to Taguchi’s design, the bacterial kinetics were tracked to determine the best conditions for biomass production (Table 1). As indicated, the nine treatments were separated into three stages based on the agitation utilized. The conditions used for the first stage (110 rpm) showed the viability of 1.0 ×10^8^ (T30:110:6.0), 3.0 × 10^8^ (T34:110:6.5), and 5.1 × 10^8^ (T38:110:7.0) CFU mL^−1^ after 48 h of incubation. For the second stage (120 rpm), the cell count was 2.5 × 10^7^ (T34:120:6.0), 5.7 × 10^8^ (T38:120:6.5), and 3.4 × 10^8^ (T30:120:7.0), and for the third stage (130 rpm), the viability was 1.6 × 10^8^ (T38:130:6.0), 5.7 × 10^9^ (T30:130:6.5), and 3.2 × 10^9^ (T34:130:7.0).

**Figure 2:**
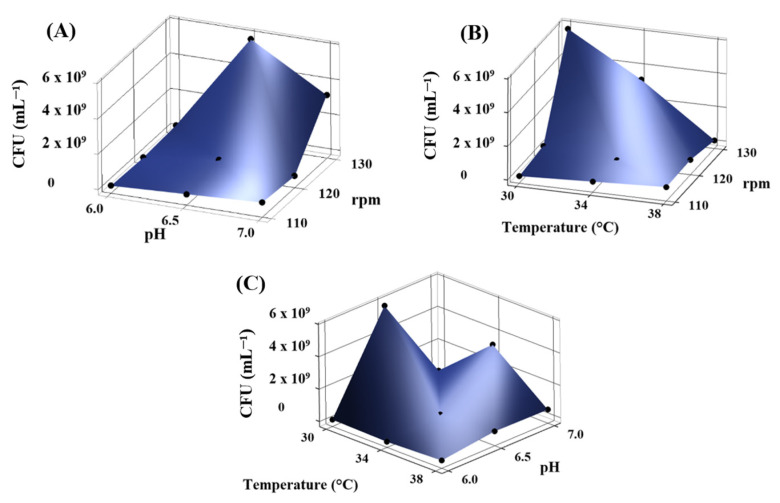
Surface response graphs for viability (CFU mL^−1^) by *Kocuria sediminis* As04 comparing (**A**) pH and rpm, (**B**) temperature and rpm, and (**C**) temperature and pH.

In Figure 2A, the combined influence of pH and agitation speed reveals that the highest CFU values are observed at pH 6.5 and 130 rpm, indicating that slightly acidic conditions and high agitation promote optimal growth for *Kocuria sediminis* As04. Figure 2B highlights the effect of temperature and agitation speed, with maximum microbial biomass recorded at 30 °C and 130 rpm. Finally, Figure 2C shows that microbial proliferation is favored at 30 °C and pH 6.5, pointing out the importance of moderate temperature and slightly acidic pH in enhancing biomass growth. The best conditions for obtaining the highest biomass were agitation at 130 rpm, 30 °C, and a pH of 6.5 (5.7 × 10^9^ CFU mL^−1^), followed by 130 rpm, pH 7.0, and 34 °C (3.2 × 10^9^ CFU mL^−1^).

Table 2 presents the effects of varying temperature, agitation speed, and pH on *Kocuria sediminis* AS04 biomass production (as dry weight) over incubation periods of 0, 4, 8, 12, 24, and 48 h. Biomass increased progressively in all treatments with time, indicating active microbial growth. Furthermore, all treatments showed significant differences from the normal growth conditions (30 °C, 100 rpm, and pH 6.9) used for *Kocuria sediminis* AS04.

The highest biomass was observed under the condition T30:130:6.5 (13.30 g L^−1^ at 48 h), followed closely by T38:130:6.0 (12.52 g L^−1^), highlighting the positive influence of high agitation (130 rpm) combined with slightly acidic to neutral pH levels. However, these treatments do not present significant differences with treatment T38:110:7.0 (10.75 g L^−1^), T34:120:6.0 (11.57 g L^−1^), and T34:130:7.0 (14.44 g L^−1^), indicating that the high temperatures (34 and 38 °C) could be an important factor for *Kocuria sediminis* AS04 biomass increase. This observation aligns with reports indicating that some *Kocuria* species exhibit thermotolerance and enhanced metabolic activity under moderately high temperatures, which can accelerate enzymatic processes and cellular division rates [44]. The variable temperature may also improve membrane fluidity and nutrient uptake efficiency, further enhancing biomass [45].

**Figure 3:**
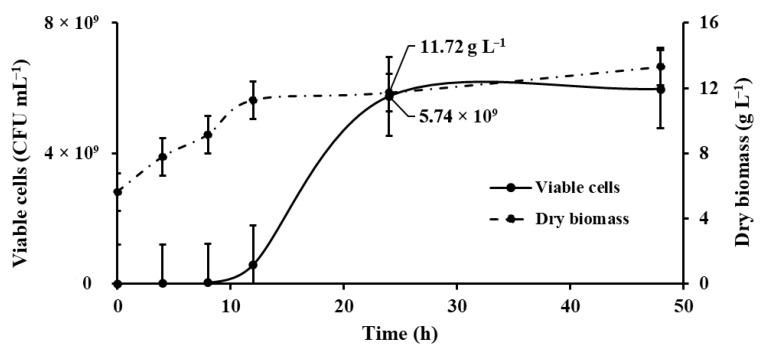
Growth kinetics showing the viable cells (CFU mL^−1^) and dry biomass (g L^−1^) with the best treatment for biomass production, *Kocuria sediminis* As04 (T30:130:6.5). The solid line represents the growth curve based on viable cells. The dashed line shows biomass accumulation as dry weight over time. Bars indicate standard deviation.

On the other hand, treatments T38:110:7.0, T38:110:6.0, and T30:130:6.5 exhibited biomass increases of 2.67, 2.54, and 2.12 g L^−1^, respectively, after 12 h of incubation, unlike the other treatments, in which the most significant biomass production occurred at 24 and 48 h, where the stationary phase was observed (Figure 3).

Overall, treatments with higher agitation speeds consistently outperformed those at 110 and 120 rpm, suggesting that enhanced oxygen transfer and mixing contribute to significantly increased biomass accumulation. Although the optimal pH varied slightly across conditions, pH 6.5 was frequently associated with high-performing treatments. These findings highlight the importance of carefully optimizing environmental parameters, particularly agitation speed and pH, to maximize *Kocuria sediminis* growth. Agitation was reported to improve the distribution of oxygen and nutrients in the bacterial broth. With moderate agitation (150 rpm), the oxygen is homogeneously distributed, with limited dead zones at the bottom [46].

The relationship between dry biomass production and viable cells (CFU mL^−1^) is presented in a graph that compares both parameters using the kinetics of the treatment with the highest biomass production (T30:130:6.5) (Figure 3).

Initially, viable cells (CFU counts) remained low up to 12 h, after which a sharp increase was observed (early logarithmic phase), reaching a maximum concentration of 5.74 × 10^9^ CFU mL^−1^ at 24 h. In contrast, biomass (dry weight) continuously increased throughout the incubation, reaching 11.72 g L^−1^ at 24 h. After this point, CFU counts and biomass showed minimal variation, suggesting that the culture reached the stationary phase. Overall, the results demonstrate active microbial growth with stabilization after 24 h, correlating CFU counts with biomass accumulation during culture.

After 24 h, both parameters plateau, with only slight fluctuations observed up to 48 h, indicating the culture has reached the stationary phase. Stabilizing viable cell counts at high densities and maintaining dry biomass concentration support the idea of metabolic equilibrium, where nutrient depletion or accumulation of inhibitory metabolites could limit further growth [47]. Overall, the data demonstrate efficient biomass production and high cell viability up to 24 h, highlighting this time point as optimal for harvesting in bioprocess applications.

For this strain, the lag phase lasts approximately 8 h, followed by a rapid exponential growth phase that peaks at 24 h. Beyond this point, the culture enters the stationary phase, maintaining its maximum biomass concentration through to the end of the observation period. The specific growth rate calculated for the kinetics of the treatment T30:130:6.5 was µ_max_ = 0.191 (h^−1^), while its time to double the biomass is t_d_ = 3.629 (h).

The growth and biomass production of bacteria are significantly influenced by environmental factors, especially temperature, pH, and agitation speed. These parameters are critical for optimizing industrial bioprocesses, as they directly affect cellular metabolism, enzymatic activity, and overall microbial productivity [48]. The findings of this study align with previous research on the growth requirements of the genus *Kocuria*. According to Bala et al. [5], *K. sediminis* strains can tolerate a relatively broad range of temperatures (25 °C to 37 °C) and pH levels (6.0 to 10.0), reflecting their origin from dynamic marine environments.

*Kocuria rosea*, isolated from hot spring environments, shows optimal growth at temperatures exceeding 40 °C, highlighting the genus’s ability to adapt to a wide temperature range [49]. This adaptability is further supported by genomic studies on related species like *Kocuria* spp., which reveal genes involved in heat stress responses, such as chaperones and proteases [50].

The growth of *Kocuria* species is significantly influenced by temperature, affecting both their specific growth rate and maximum biomass accumulation, typically measured by OD_600_. For example, *Kocuria rosea* cultivated at 40 °C with agitation at 75 rpm demonstrated a specific growth rate of 0.17 h^−1^, achieving a maximum biomass of 3.1 g L^−1^ after 36 h [51]. In another study, *K. rosea* displayed a lag phase of approximately 3 h during batch fermentation, followed by an exponential phase, reaching a specific growth rate of 0.108 h^−1^. After 20 h, the culture achieved a maximum biomass concentration of 2.7 g L^−1^, indicating rapid adaptation and efficient biomass production under optimized conditions tested [52]. Mitra et al. [53] found a specific growth rate (μ_max_) of *K. marine* in a culture growth with glucose at 0.2538 h^−1^ and with maltose at 0.118 h^−1^, a lag phase of 6 h for both substrates, followed by an exponential phase that continued up to 24 h, where the stationary phase was set up, with a maximum biomass of 6 g L^−1^. The present work reported a specific growth rate (0.19 h^−1^) in other *Kocuria* species. However, biomass production was higher (13.3 g L^−1^) than that reported in other species. Similarly, strains of *Kocuria salsicia* isolated from cheese brine exhibited wide thermal adaptability, growing across a range of 5–42 °C. Notably, strains KS6 and KS8 maintained growth even at 5 °C, while at 25 °C and 30 °C, they entered the stationary phase after approximately 60 h, achieving higher OD values compared to the control strain *Staphylococcus aureus* ATCC 29213 [54]. These findings collectively underscore the considerable temperature tolerance and adaptability of *Kocuria* species, positioning them as promising candidates for diverse biotechnological applications that require robust performance under variable environmental conditions [55].

### 3.3. Total Pigments

**Figure 4:**
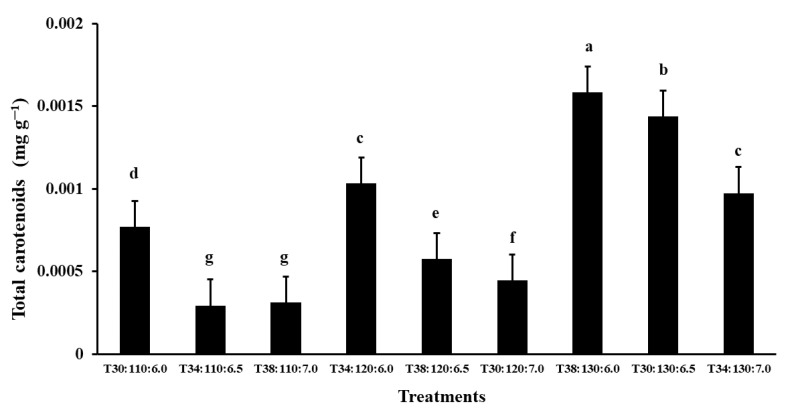
The concentration of total carotenoids from *Kocuria sediminis* As04 at 48 h of culture. Treatment codes indicate temperature (°C), agitation speed (rpm), and pH value. Bars represent the standard error. Different lowercase letters indicate significant differences between treatments. Means with different letters are significantly different. Tukey’s test was used with a significance level of *p* < 0.05.

The total carotenoid content showed statistically significant variation among the Taguchi treatments (*p* < 0.05). Treatment T38:130:6.0 exhibited the highest carotenoid concentration (0.0016 mg g^−1^), followed by treatment T30:130:6.5 (0.0014 mg g^−1^), with a significant difference between them (Figure 4). Both treatments also presented significantly higher carotenoid levels than the remaining treatments, indicating their effectiveness in promoting carotenoid accumulation under the tested conditions. The second treatment had the highest biomass production.

The treatments with a pH of 6.0 (T30:110:6.0, T34:120:6.0, and T38:130:6.0), conducted under varying temperatures and agitation speeds (rpm), exhibited the highest carotenoid concentrations. These results suggest that a lower pH may be key in enhancing pigment production in *Kocuria sediminis* AS04, potentially independent of the effects of temperature and agitation conditions. Ramesh et al. [56] reported that microbial pigments such as melanin, riboflavin, violacein, flexirubin, pyocyanin, carotenoids, and prodigiosin are typically produced by genera such as *Bacillus, Chromobacterium, Chryseobacterium, Pseudomonas, Serratia, and Streptomyces* under specific environmental conditions (22–28 °C, pH 5–6, and 100–150 rpm).

Extremophilic bacteria adapted to live in such niches produce pigments as a crucial survival mechanism and adaptive response under these conditions. Many bacterial pigments, especially carotenoids (which give yellow, orange, and red colors), are potent antioxidants. By producing more pigments, bacteria can neutralize free radicals and protect themselves from oxidative damage caused by extreme conditions such as high temperature and acidic pH [57].

Species from the family *Micrococcaceae*, particularly those belonging to the genus *Kocuria,* have demonstrated notable potential for carotenoid pigment production under various cultivation conditions. For example, *Kocuria* spp. strain QWT-12 primarily produced neurosporene and violaxanthin when cultured in TSB supplemented with 3% NaCl at 37 °C. Both pigments had significant anticancer activity [58]. Similarly, *Kocuria marina* DAG II, isolated by Mitra et al. [53], was reported to produce β-cryptoxanthin at a concentration of 0.0025 mg g^−1^. A similar result was obtained in the present work by applying the best treatment, T38:130:6.0, with a concentration of 0.0016 mg g^−1^ total carotenoid pigment.

Further highlighting the carotenoid diversity within this genus, *Kocuria* spp. GMA was shown to synthesize multiple carotenoids, including lycopene, β-cryptoxanthin, and a novel compound named kocumarin, displaying potent antioxidant, antimicrobial, and antibiofilm activities [28]. In addition, *Kocuria flava* SIF3 produces a yellow carotenoid pigment with a maximum absorbance at 437 nm when grown in nutrient broth, exhibiting significant antioxidant and antimicrobial effects [59]. Likewise, *Kocuria palustris* isolates FT-7.22 and FT-5.12 can synthesize sarcinaxanthin, a rare C_50_ carotenoid, with enhanced yields under increased aeration, agitation, and light exposure. These reports demonstrated the antioxidant and photoprotective activities of *Kocuria* pigments, further highlighting the adaptability of *Kocuria* species to different environmental conditions [18]. These findings also emphasize the industrial potential of *Kocuria* species as natural pigment producers for pharmaceutical, cosmetic, and food applications.

Statistical analysis revealed that dry biomass production was significantly affected by temperature, agitation, and pH (*p* ≤ 0.002), as well as by the interactions between temperature × agitation and temperature × pH (*p* < 0.05), indicating that both individual factors and their synergistic effects played a critical role in promoting growth (Table 3). In contrast, viable cell counts (CFU) remained unaffected across all tested conditions (*p* > 0.2), suggesting that variations in biomass likely reflect changes in cell mass or extracellular material rather than cell number. Regarding pigment production, agitation (*p* = 0.023) emerged as a key modulator, likely due to its influence on oxygen transfer and shear conditions, with a highly significant effect observed for the agitation × pH interaction (*p* = 0.003). Additionally, the three-way interaction (temperature × agitation × pH) showed a marginal effect (*p* = 0.05), implying that pigment biosynthesis is sensitive to the combined influence of multiple environmental parameters.

Some studies have demonstrated the significant influence of temperature, agitation, and pH on bacterial growth. A study on *Clostridium acetobutylicum, Clostridium beijerinckii,* and *Clostridium saccharoperbutylacetonicum* revealed that these three factors mutually impact both growth and secondary metabolites, such as butanol production, with optimal conditions varying by strain [60]. Similarly, Coleman et al. [61] investigated *Escherichia coli* O157:H7 and found that agitation, pH, and inoculum density significantly affected growth dynamics, particularly at lower temperatures.

Optimizing microbial pigment production often requires a multifactorial approach involving nutritional and physicochemical parameters. Nguyen et al. [62] reported an efficient prodigiosin pigment production process using *Serratia marcescens* TKU011, marine α-chitin, and casein as carbon and nitrogen sources. Under bioreactor conditions, they achieved up to 6.2 g L^−1^ yields in 8 h. Key factors included a slightly acidic pH (5.65–6.15), a moderate temperature (25–27.5 °C), and the addition of inorganic salts, such as K_2_HPO_4_ and CaSO_4_. In contrast, the highest carotenoid yield obtained in *Kocuria sediminis* in our study was 0.0016 mg g^−1^, produced at 38 °C and pH 6.0. This suggests that, although biosynthetic pathways and environmental responses differ between strains and pigment classes, acidification and temperature stress may similarly activate pigment biosynthesis. These findings support the notion that optimizing culture conditions is essential to maximizing pigment yields and that investigating nutrient formulations and bioreactor scaling could significantly enhance carotenoid production in actinobacteria.

Recent studies have demonstrated that agro-industrial waste streams can efficiently serve as substrates for microbial pigment production, representing nitrogen sources. Additionally, with mineral salt supplementation, it is possible to enhance pigment yields [63].

Recent advances in microbial carotenoid production have highlighted the potential of actinobacteria to produce pigments efficiently under optimized conditions. Hegazy et al. [64] reported using cheese whey as a low-cost substrate for *Micrococcus luteus* ATCC 9341 and achieving a carotenoid yield of 2.19 g L^−1^ through Box–Behnken design optimization. The critical parameters of whey concentration, pH, temperature, inoculum size, and agitation were systematically adjusted, resulting in high productivity (0.045 g L^−1^ h^−1^) and the identification of 12 carotenoids.

However, one of the main limitations in the production of pigments is the reduced oxygen transfer efficiency in larger bioreactors, which may impair pigment biosynthesis, as *Kocuria* often requires aerobic conditions for optimal production [65]. Additionally, the cells may be sensitive to high shear stress generated by industrial mixing systems, potentially affecting viability or pigment output [66]. Maintaining uniform pH, temperature, and nutrient distribution also becomes more difficult at scale, leading to gradients that can disrupt metabolic activity [48].

### 3.4. Determination of Pigments from Kocuria sediminis Biomass by MS/MS

**Figure 5:**
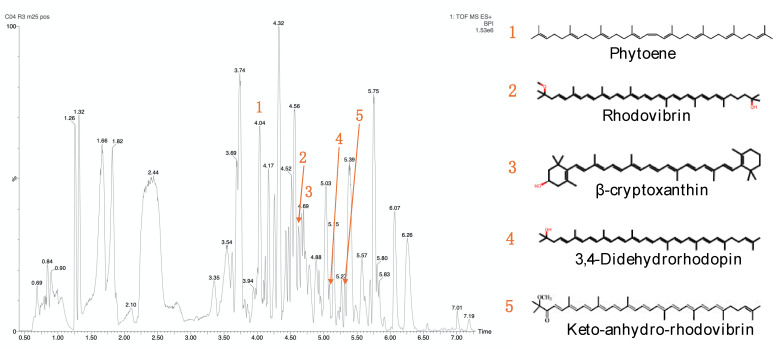
Base peak intensity (BPI) chromatogram in positive mode and chemical structures of carotenoid compounds found in cell extracts from *Kocuria sediminis* As04 obtained under better culture optimization conditions.

Key carotenoid compounds were identified from the extract obtained from *Kocuria sediminis* As04, such as Phytoene, β-cryptoxanthin, Rhodovibrin, 3,4-didehydrorhodopin, and Keto-anhydro-rhodovibri (Figure 5, Table 4). Other *Kocuria* species have been reported to produce β-cryptoxanthin [28,53] and to present phytoene [42].

The first colorless carotenoid in the microbial biosynthetic pathway is phytoene, a precursor of neurosporene or lycopene [67]. Phytoene has been reported in other *Kocuria* cell extracts with *m*/*z* 545.4 [M + H]^+^, while rhodovibrin was found at *m*/*z* 583.5 [M + H]^+^ [42]. This is similar the experimental mass found in the present work for those pigments produced by *Kocuria sediminis* As04.

A monohydroxy carotenoid, β-cryptoxanthin, acts as an intermediate in the biosynthesis of the dihydroxy carotenoids, such as zeaxanthin [53]. β-Cryptoxanthin is a xanthophyll carotenoid with the potential to act as provitamin A and has been reported to improve bone health [28,53]. These results show the natural production of β-cryptoxanthin by bacterial strains, presenting potential opportunities for scaling up and improving cultivation techniques to increase β-cryptoxanthin production. The 3,4-didehydrorhodopin is a carotenol with a rhodopin structure, with two hydrogen atoms abstracted from the C(3)–C(4) bond to form an extra trans double bond. It has a role as a bacterial metabolite and was earlier reported in *Rhodomicrobium vannielii* [68].

The 3,4-didehydrorhodopin and Keto-anhydro-rhodovibri are precursors of spirilloxanthin in carotenoid biosynthesis [69], as can be observed in KEGG (https://www.kegg.jp/pathway/map00906, accessed on 1 July 2025). These compounds have been reported in photosynthetic bacteria such as the genera *Rhodospirillum* and *Rhodobacter* [69,70,71].

### 3.5. Characterization of Kocuria sediminis AS04 Biomass by SEM and FTIR

**Figure 6:**
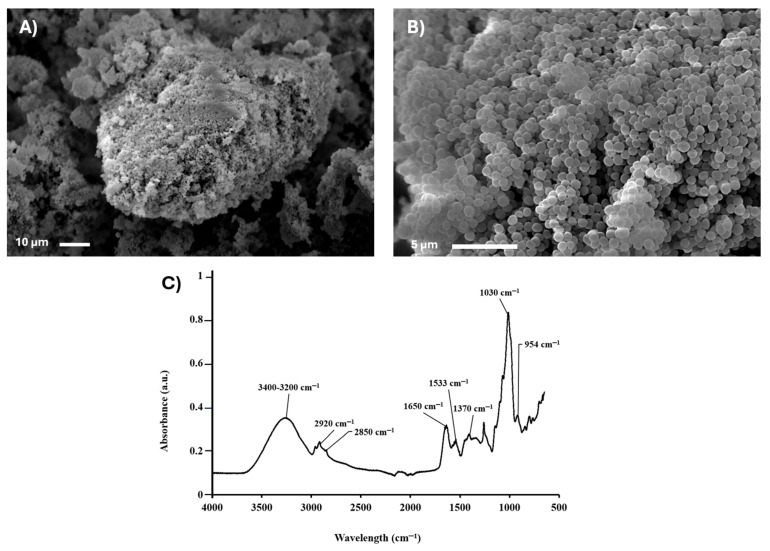
Structural characterization of *Kocuria sediminis* AS04 by SEM: (**A**) 1000× and (**B**) 5000×; (**C**) cellular spectrum by FTIR.

A micrograph of *Kocuria sediminis* As04 biomass showed a large, irregularly shaped agglomerate form. The surface appears rough and heterogeneous, indicating a dense aggregation of cocci (Figure 6A). The close cell arrangement may also indicate active growth [72].

Another micrograph taken at 5000× (Figure 6B) displayed a compact and uniform distribution of spherical, coccoid-shaped particles, likely representing individual bacterial cells. The SEM determined cocci between 0.5 and1.2 µm. The high degree of size and shape uniformity and tight packing suggests a pure culture of coccoid bacteria. This morphology is characteristic of Gram-positive cocci, such as *Kocuria* spp. [73], aligning with previous identification [9,74].

The observed compact morphology is consistent with characteristics reported for bacteria with high surface-area-to-volume ratios, which are generally associated with efficient nutrient uptake and potential resilience to environmental fluctuations [75]. These morphological traits, documented through SEM analysis, contribute valuable baseline data for strain characterization and support the phylogenetic placement of the isolate within its expected taxonomic group.

*Kocuria* spp. thrive in diverse and sometimes extreme environments, including soils, sediments, and clinical settings [76]. Their survival is partly attributed to their compact morphology, which supports efficient nutrient uptake (via a high surface-area-to-volume ratio), desiccation resistance, and UV tolerance [65]. The morphological features shown in the SEM images support these ecological traits and reinforce the identification of the isolate as *Kocuria sediminis* AS04.

The FTIR analysis of *Kocuria sediminis* As04 biomass revealed key functional group vibrations associated with cellular components and intracellular carotenoid pigments. The spectra showed typical bacterial fingerprint features, along with distinct signals attributed to carotenoids, indicating their biosynthesis and accumulation within the cells (Figure 6C).

The presence of carotenoids was supported by a 1650 cm^−1^ band corresponding to the C=C stretching vibrations of conjugated double bonds, an essential structural element of carotenoid pigments [77]. This peak is typically strong and sharp, reflecting the extended polyene chain structure. Additionally, a band at 1370 cm^−1^ was assigned to CH_2_ and CH_3_ bending vibrations, further supporting the aliphatic nature of the pigment side chains [78].

Signals around 2920 and 2850 cm^−1^ represented the asymmetric and symmetric stretching of aliphatic –CH_2_ and –CH_3_ groups, consistent with the hydrocarbon backbone of carotenoids [79]. A smaller peak near 954 cm^−1^ was observed, indicative of trans-configuration C–H out-of-plane bending, a characteristic feature of carotenoids such as lycopene or β-carotene [80].

In parallel, the broader spectrum of *Kocuria* cells exhibited features standard to Gram-positive bacteria. A broad band at 3300–3400 cm^−1^ was attributed to O–H and N–H stretching, reflecting contributions from hydroxyl groups in protein polysaccharides and amide groups [81]. The amide I (around 1650 cm^−1^) and amide II (around 1533 cm^−1^) bands were present, indicating protein content within the cellular matrix [82].

The region 1030 cm^−1^ showed strong signals corresponding to C–O and C–N stretching vibrations, possibly arising from the cell wall’s peptidoglycan, nucleic acids, and polysaccharide components [83]. These characteristics confirm the presence of proteins, lipids, and polysaccharides typically found in bacterial cell walls, reinforcing the SEM findings. These data highlight the morphological and structural features that enable *Kocuria sediminis* AS04 to adapt and thrive in diverse environments while validating its taxonomic classification within the genus *Kocuria*. Apart from their industrial relevance, these pigments protect the organism against oxidative stress and UV radiation [42].

## 4. Conclusions

This study demonstrates that *Kocuria sediminis* AS04 exhibits the potential for pigment production and biomass accumulation. The total carotenoid pigment was optimized at 38 °C, 130 rpm, and 6.0 pH, suggesting that this strain can adapt to extreme conditions, and pigment production is increased as an adaptive mechanism. MS/MS analysis tentatively identified carotenoids such as β-cryptoxanthin, rhodovibrin, 3,4-didehydrorhodopin, and keto-anhydro-rhodovibrin, compounds typically associated with pigmentation in bacteria. The detection of phytoene, a key precursor in the carotenoid biosynthetic pathway, indicated active pigment production within the strain. These findings underscore the biotechnological potential of *Kocuria sediminis* AS04 as a source of natural pigments. Nevertheless, further research is needed to address the scalability of pigment production, evaluate pigment stability and functionality under industrial conditions, and explore practical applications in sectors such as textiles, food, cosmetics, and pharmaceuticals. Optimizing culture strategies or applying metabolic engineering could also enhance pigment yields and expand the diversity of bioactive compounds produced by this strain.

## Figures and Tables

**Table 1:** Taguchi’s experimental design included three different temperatures, stirring speeds, and pH combinations.

Treatment Code	Temperature (°C)	Stirring Speed (rpm)	pH
T30:110:6.0	30	110	6.0
T34:110:6.5	34	110	6.5
T38:110:7.0	38	110	7.0
T34:120:6.0	34	120	6.0
T38:120:6.5	38	120	6.5
T30:120:7.0	30	120	7.0
T38:130:6.0	38	130	6.0
T30:130:6.5	30	130	6.5
T34:130:7.0	34	130	7.0

Each treatment was conducted in triplicate.

**Table 2:** Dry biomass (g L^−1^) was produced during the different treatments at different culture times of *Kocuria sediminis* As04.

Treatments	Dry Biomass (g L^−1^) *					
	T0	T4	T8	T12	T24	T48
NC	5.14 ± 0.25 ^Db^	6.24 ± 0.07 ^Ca^	6.59 ± 0.16 ^BCa^	7.1 ± 0.24 ^Bc^	8.64 ± 0.41 ^Ad^	8.71 ± 0.32 ^Ac^
T30:110:6.0	7.53 ± 0.19 ^Ba^	8.22 ± 0.8 ^Aa^	8.45 ± 1.06 ^Aa^	9.39 ± 0.68 ^Aabc^	9.54 ± 0.16 ^Acd^	10.13 ± 1.02 ^Abc^
T34:110:6.5	7.15 ± 0.38 ^Baab^	9.03 ± 2.1 ^Aa^	9.3 ± 1.2 ^Aa^	9.5 ± 0.32 ^Aabc^	9.58 ± 0.12 ^Acd^	10.37 ± 0.93 ^Abc^
T38:110:7.0	6.42 ± 1.02 ^Cab^	6.73 ± 0.39 ^Ba^	7.06 ± 0.93 ^Ba^	9.73 ± 0.67 ^Aabc^	10.02 ± 0.17 ^Abc^	10.75 ± 0.7 ^Aabc^
T34:120:6.0	5.74 ± 0.19 ^Dab^	6.933 ± 0.7 ^Ca^	8.83 ± 1.2 ^BCa^	9.91 ± 0.79 ^ABab^	11.35 ± 0.4 ^Aa^	11.57 ± 1.0 ^Aabc^
T38:120:6.5	6.61 ± 0.24 ^Bab^	6.9 ± 0.81 ^Aa^	8.48 ± 1.04 ^Aa^	8.74 ± 2.0 ^Aabc^	9.11 ± 0.13 ^Acd^	9.57 ± 0.82 ^Ac^
T30:120:7.0	5.79 ± 0.1.2 ^Dab^	6.72 ± 0.7 ^Ca^	7.63 ± 0.26 ^BCa^	8.29 ± 1.38 ^ABbc^	9.34 ± 0.27 ^ABcd^	9.78 ± 0.59 ^Ac^
T38:130:6.0	5.95 ± 0.65 ^Dab^	6.71 ± 1.5 ^Ca^	7.3 ± 0.58 ^Ca^	9.84 ± 0.53 ^Babc^	11.27 ± 0.20 ^ABab^	12.52 ± 0.5 ^Aab^
T30:130:6.5	5.64 ± 1.03 ^Dab^	7.8 ± 0.16 ^Ca^	9.15 ± 1.54 ^BCa^	11.27 ± 0.6 ^ABa^	11.72 ± 1.18 ^Aa^	13.3 ± 0.74 ^Aa^
T34:130:7.0	6.13 ± 1.53 ^Bab^	8.0 ± 1.2 ^Ba^	8.05 ± 1.8 ^Ba^	8.5 ± 0.41 ^ABbc^	9.3 ± 0.15 ^ABcd^	11.44 ± 1.8 ^Aabc^

* Dry weight. Capital letters compare each treatment during the 48 h of kinetics (between columns), and lowercase letters compare all the treatments at each time point (between rows). Means with different letters are significantly different. Tukey’s test was used with a significance level of *p* < 0.05. Values shown after ± correspond to the standard deviation. NC: Normal conditions (30 °C, 100 rpm, and pH 6.9).

**Table 3:** The effect of temperature, agitation, and pH on dry biomass production, viable cells, and total pigments from *Kocuria sediminis* AS04. Tukey Studentized Rank Analysis (HSD) Type III. Tukey’s Honestly Significant Difference (HSD) test was applied to determine statistically significant differences among the treatment means based on the Type III sum of squares.

Factors and Interactions	Dry Biomass	Viable Cells	Total Pigments
	*p*-Value		
Temperature	0.002	0.477	0.912
Agitation	<0.0001	0.208	0.023
pH	0.002	0.598	0.272
Temperature × Agitation	0.005	0.281	0.166
Temperature × pH	0.03	0.713	0.502
Agitation × pH	0.138	0.257	0.003
Temperature × Agitation × pH	0.084	0.479	0.05

**Table 4:** Putative identification of pigments from *Kocuria sediminis* As04 in positive mode [M + H]^+^.

Peak	Putative Identification	Retention Time (min)	Exact Mass (Da)	Experimental Mass (*m*/*z*)
1	Phytoene	4.05	544.500	545.293
2	Rhodovibrin	4.62	584.459	585.327
3	β-cryptoxanthin	4.69	552.433	553.337
4	3,4-Didehydrorhodopin	5.09	552.433	553.321
5	Keto-anhydro-rhodovibrin	5.31	580.428	581.368

## Data Availability

The raw data supporting the conclusions of this article will be made available by the authors on request.

## References

[1] Chandrasekaran M., Paramasivan M. (2024). Plant Growth-Promoting Bacterial (PGPB) Mediated Degradation of Hazardous Pesticides: A Review. Int. Biodeterior. Biodegrad..

[2] Mauceri A., Puccio G., Faddetta T., Abbate L., Polito G., Caldiero C., Renzone G., Lo Pinto M., Alibrandi P., Vaccaro E. (2024). Integrated Omics Approach Reveals the Molecular Pathways Activated in Tomato by Kocuria Rhizophila, a Soil Plant Growth-Promoting Bacterium. Plant Physiol. Biochem..

[3] Achal V., Pan X., Zhang D. (2011). Remediation of Copper-Contaminated Soil by Kocuria Flava CR1, Based on Microbially Induced Calcite Precipitation. Ecol. Eng..

[4] Bergey D.H. (2012). Bergey’s Manual ^®^ of Systematic Bacteriology.

[5] Bala M., Kaur C., Kaur I., Khan F., Mayilraj S. (2012). *Kocuria sediminis* sp. Nov., Isolated from a Marine Sediment Sample. Antonie Van Leeuwenhoek.

[6] de Souza R., Ambrosini A., Passaglia L.M.P. (2015). Plant Growth-Promoting Bacteria as Inoculants in Agricultural Soils. Genet. Mol. Biol..

[7] Uzair B., Menaa F., Khan B.A., Mohammad F.V., Ahmad V.U., Djeribi R., Menaa B. (2018). Isolation, Purification, Structural Elucidation and Antimicrobial Activities of Kocumarin, a Novel Antibiotic Isolated from Actinobacterium Kocuria Marina CMG S2 Associated with the Brown Seaweed Pelvetia Canaliculata. Microbiol. Res..

[8] Dif G., Belaouni H.A., Goudjal Y., Yekkour A., Djemouai N., Zitouni A. (2021). Potential for Plant Growth Promotion of Kocuria Arsenatis Strain ST19 on Tomato under Salt Stress Conditions. S. Afr. J. Bot..

[9] Guardado-Fierros B.G., Tuesta-Popolizio D.A., Lorenzo-Santiago M.A., Rodriguez-Campos J., Contreras-Ramos S.M. (2024). Comparative Study between Salkowski Reagent and Chromatographic Method for Auxins Quantification from Bacterial Production. Front. Plant Sci..

[10] Venil C.K., Zakaria Z.A., Ahmad W.A. (2013). Bacterial Pigments and Their Applications. Process Biochem..

[11] Rather L.J., Mir S.S., Ganie S.A., Shahid-ul-Islam, Li Q. (2023). Research Progress, Challenges, and Perspectives in Microbial Pigment Production for Industrial Applications—A Review. Dye. Pigment..

[12] Mendes A.R., Spínola M.P., Lordelo M., Prates J.A.M. (2024). Advances in Bioprocess Engineering for Optimising Chlorella Vulgaris Fermentation: Biotechnological Innovations and Applications. Foods.

[13] Grewal J., Woła̧cewicz M., Pyter W., Joshi N., Drewniak L., Pranaw K. (2022). Colorful Treasure From Agro-Industrial Wastes: A Sustainable Chassis for Microbial Pigment Production. Front. Microbiol..

[14] Xu S., Gao S., An Y. (2023). Research Progress of Engineering Microbial Cell Factories for Pigment Production. Biotechnol. Adv..

[15] Harshini P., Varghese R., Pachamuthu K., Ramamoorthy S. (2025). Enhanced Pigment Production from Plants and Microbes: A Genome Editing Approach. 3 Biotech.

[16] Dasgupta Mandal D., Majumdar S. (2023). Bacteria as Biofactory of Pigments: Evolution beyond Therapeutics and Biotechnological Advancements. J. Biosci. Bioeng..

[17] Molina A.K., Corrêa R.C.G., Prieto M.A., Pereira C., Barros L. (2023). Bioactive Natural Pigments’ Extraction, Isolation, and Stability in Food Applications. Molecules.

[18] Mendes-Silva T.d.C.D., Vidal E.E., de Souza R.d.F.R., Schmidt K.d.C., Mendes P.V.D., da Silva Andrade R.F., da Silva Oliveira F.G., de Lucena B.T.L., de Oliveira M.B.M., dos Santos Correia M.T. (2021). Production of Carotenoid Sarcinaxanthin by Kocuria Palustris Isolated from Northeastern Brazil Caatinga Soil and Their Antioxidant and Photoprotective Activities. Electron. J. Biotechnol..

[19] Zia-Ul-Haq M., Dewanjee S., Riaz M. (2021). Carotenoids: Structure and Function in the Human Body.

[20] Sundararajan P., Ramasamy S.P. (2024). Current Perspectives on Industrial Application of Microbial Carotenoid as an Alternative to Synthetic Pigments. Sustain. Chem. Pharm..

[21] Brahma D., Dutta D. (2022). Antioxidant Property of Beta-Cryptoxanthin Produced by Kocuria Marina DAGII. Mater. Today Proc..

[22] Kumar D., Kumar A., Sharma J. (2016). Degradation Study of Lindane by Novel Strains *Kocuria* sp. DAB-1Y and *Staphylococcus* sp. DAB-1W. Bioresour. Bioprocess..

[23] Kulkarni V.M., Dixit A.S., Patwardhan A.V., Bajwa A.S. (2018). A Different Approach to Augment Pigment Production and Its Extraction from Kocuria Flava by Using Ultrasound Technique. J. Biol. Act. Prod. Nat..

[24] Ashraf Y.Z.K. (2017). Degradation of Diesel-Oil by a Newly Isolated *Kocuria sediminis* DDK6. Afr. J. Microbiol. Res..

[25] Khalifa A.Y.Z. (2017). Scanning Electron Microscopy and Antibiotic Sensitivity of the Actinobacterium, *Kocuria sediminis* DDK6. J. Appl. Biol. Biotechnol..

[26] Yabuzaki J. (2017). Carotenoids Database: Structures, Chemical Fingerprints and Distribution among Organisms. Database.

[27] Ambati R., Phang S.-M., Ravi S., Aswathanarayana R. (2014). Astaxanthin: Sources, Extraction, Stability, Biological Activities and Its Commercial Applications—A Review. Mar. Drugs.

[28] Ibrahim G.S., El-Shall F.N., Arafa A.A., Shalabi A., El Awady M.E. (2024). Bio-Production and Characterization of Carotenoid Yellow Pigment from *Kocuria* sp. GMA and Exploring Its Sustainable Antioxidant, Antimicrobial and Antibiofilm Properties. Egypt. J. Chem..

[29] Britton G. Functions of Intact Carotenoids. Carotenoids.

[30] Krinsky N.I., Johnson E.J. (2005). Carotenoid Actions and Their Relation to Health and Disease. Mol. Asp. Med..

[31] Guardado-Fierros B.G., Lorenzo-Santiago M.A., Kirchmayr M.R., Patrón-Soberano O.A., Rodriguez-Campos J., Contreras-Ramos S.M. (2025). Biocontrol and Plant Growth-Promoting Activities of Airborne Bacteria. World J. Microbiol. Biotechnol..

[32] Tuesta-Popolizio D.A., Velázquez-Fernández J.B., Rodriguez-Campos J., Contreras-Ramos S.M. (2021). Isolation and Identification of Extremophilic Bacteria with Potential as Plant Growth Promoters (Pgpb) of A Geothermal Site: A Case Study. Geomicrobiol. J..

[33] Gutiérrez-Santa Ana A., Carrillo-Cerda H.A., Rodriguez-Campos J., Kirchmayr M.R., Contreras-Ramos S.M., Velázquez-Fernández J.B. (2020). Volatile Emission Compounds from Plant Growth-Promoting Bacteria Are Responsible for the Antifungal Activity against *F. solani*. 3 Biotech.

[34] (2020). Standard Test Methods for Moisture, Ash, and Organic Matter of Peat and Other Organic Soils.

[35] Zhi R., Yang A., Zhang G., Zhu Y., Meng F., Li X. (2019). Effects of Light-Dark Cycles on Photosynthetic Bacteria Wastewater Treatment and Valuable Substances Production. Bioresour. Technol..

[36] Fish W.W., Perkins-Veazie P., Collins J.K. (2002). A Quantitative Assay for Lycopene That Utilizes Reduced Volumes of Organic Solvents. J. Food Compos. Anal..

[37] Zhang J., Wang P., Long H., Su S., Wu Y., Wang H. (2022). Metabolomics Analysis Reveals the Physiological Mechanism Underlying Growth Restriction in Maize Roots under Continuous Negative Pressure and Stable Water Supply. Agric. Water Manag..

[38] Liu P.P., Shan G.S., Zhang F., Chen J.N., Jia T.Z. (2018). Metabolomics Analysis and Rapid Identification of Changes in Chemical Ingredients in Crude and Processed Astragali Radix by UPLC-QTOF-MS Combined with Novel Informatics UNIFI Platform. Chin. J. Nat. Med..

[39] Vannabhum M., Ziangchin N., Thepnorarat P., Akarasereenont P. (2023). Metabolomic Analysis of Thai Herbal Analgesic Formula Based on Ultra-High-Performance Liquid Chromatography-Quadrupole Time-of-Flight Mass Spectrometry. Heliyon.

[40] Hou Y.F., Bai L., Guo S., Hu J.B., Zhang S.S., Liu S.J., Zhang Y., Li S., Ho C.T., Bai N.S. (2023). Nontargeted Metabolomic Analysis of Four Different Parts of Actinidia Arguta by UPLC-Q-TOF-MSE. Food Res. Int..

[41] Metwally R.A., El-Sersy N.A., El Sikaily A., Sabry S.A., Ghozlan H.A. (2022). Optimization and Multiple in Vitro Activity Potentials of Carotenoids from Marine *Kocuria* sp. RAM1. Sci. Rep..

[42] Alam T., Din S.U., Abdullah M., Ali M., Badshah M., Farman M., Khan S., Hasan F., Shah A.A. (2025). Bioactive Metabolites from Radioresistant Bacterium *Kocuria* sp. TMM 11 and Their Role in Prevention of Ultraviolet-Induced Photodamages. Curr. Microbiol..

[43] Gashi N., Szőke Z., Fauszt P., Dávid P., Mikolás M., Gál F., Stündl L., Remenyik J., Paholcsek M. (2025). Bioaerosols in Agriculture: A Comprehensive Approach for Sustainable Crop Health and Environmental Balance. Agronomy.

[44] Quadri S.R. (2024). Survival Strategy, Metabolic Potential and Taxonomic Reframe of *Kocuria polaris*. J. Pure Appl. Microbiol..

[45] Nedwell D.B. (1999). Effect of Low Temperature on Microbial Growth: Lowered Affinity for Substrates Limits Growth at Low Temperature. FEMS Microbiol. Ecol..

[46] Mulakhudair A.R., Al-Mashhadani M.K.H., Kokoo R. (2022). Tracking of Dissolved Oxygen Distribution and Consumption Pattern in a Bespoke Bacterial Growth System. Chem. Eng. Technol..

[47] Boer V.M., Crutchfield C.A., Bradley P.H., Botstein D., Rabinowitz J.D. (2010). Growth-Limiting Intracellular Metabolites in Yeast Growing under Diverse Nutrient Limitations. Mol. Biol. Cell.

[48] Ortiz-Cortés L.Y., Ventura-Canseco L.M.C., Abud-Archila M., Ruíz-Valdiviezo V.M., Velázquez-Ríos I.O., Alvarez-Gutiérrez P.E. (2021). Evaluation of Temperature, PH and Nutrient Conditions in Bacterial Growth and Extracellular Hydrolytic Activities of Two *Alicyclobacillus* spp. Strains. Arch. Microbiol..

[49] Kim S.-H., Kim W.-J., Ryu J., Yerefu Y., Tesfaw A. (2025). Amylase Production by the New Strains of *Kocuria rosea* and *Micrococcus endophyticus* Isolated from Soil in the Guassa Community Conservation Area. Fermentation.

[50] Mousa W.K., Abu-Izneid T., Salah-Tantawy A. (2024). High-Throughput Sequencing Reveals the Structure and Metabolic Resilience of Desert Microbiome Confronting Climate Change. Front. Plant Sci..

[51] Vidal L., Christen P., Coello M.N. (2000). Feather Degradation by Kocuria Rosea in Submerged Culture. World J. Microbiol. Biotechnol..

[52] Bertsch A., Coello N. (2005). A Biotechnological Process for Treatment and Recycling Poultry Feathers as a Feed Ingredient. Bioresour. Technol..

[53] Mitra R., Chaudhuri S., Dutta D. (2017). Modelling the Growth Kinetics of *Kocuria marina* DAGII as a Function of Single and Binary Substrate during Batch Production of Β-Cryptoxanthin. Bioprocess Biosyst. Eng..

[54] Youn H.-Y., Seo K.-H. (2022). Isolation and Characterization of Halophilic *Kocuria salsicia* Strains from Cheese Brine. Food Sci. Anim. Resour..

[55] Timkina E., Drábová L., Palyzová A., Řezanka T., Maťátková O., Kolouchová I. (2022). *Kocuria* Strains from Unique Radon Spring Water from Jachymov Spa. Fermentation.

[56] Ramesh C., Prasastha V.R., Venkatachalam M., Dufossé L. (2022). Natural Substrates and Culture Conditions to Produce Pigments from Potential Microbes in Submerged Fermentation. Fermentation.

[57] Kochhar N., Kavya I.K., Shrivastava S., Ghosh A., Rawat V.S., Sodhi K.K., Kumar M. (2022). Perspectives on the Microorganism of Extreme Environments and Their Applications. Curr. Res. Microb. Sci..

[58] Rezaeeyan Z., Safarpour A., Amoozegar M.A., Babavalian H., Tebyanian H., Shakeri F. (2017). High Carotenoid Production by a Halotolerant Bacterium, *Kocuria* sp. Strain QWT-12 and Anticancer Activity of Its Carotenoid. EXCLI J..

[59] Mal S.A., Ibrahim G.S., Al Khalaf M.I., Al-Hejin A.M., Bataweel N.M., Abu-Zaid M. (2022). Production and Partial Characterization of Yellow Pigment Produced by *Kocuria flava* Isolate and Testing Its Antioxidant and Antimicrobial Activity. Int. J. Life Sci. Pharma Res..

[60] Feldmane L., Raita S., Berzina I., Geiba Z., Mika T., Kuzmika I., Spalvins K. (2024). Effects of Temperature, PH, and Agitation on Growth and Butanol Production of *Clostridium Acetobutylicum*, *Clostridium Beijerinckii*, and *Clostridium Saccharoperbutylacetonicum*. Environ. Clim. Technol..

[61] Coleman M.E., Tamplin M.L., Phillips J.G., Marmer B.S. (2003). Influence of Agitation, Inoculum Density, PH, and Strain on the Growth Parameters of *Escherichia coli* O157:H7—Relevance to Risk Assessment. Int. J. Food Microbiol..

[62] Nguyen V.B., Chen S.-P., Nguyen T.H., Nguyen M.T., Tran T.T.T., Doan C.T., Tran T.N., Nguyen A.D., Kuo Y.-H., Wang S.-L. (2019). Novel Efficient Bioprocessing of Marine Chitins into Active Anticancer Prodigiosin. Mar. Drugs.

[63] Nguyen T.-H., Wang S.-L., Nguyen D.-N., Nguyen A.-D., Nguyen T.-H., Doan M.-D., Ngo V.-A., Doan C.-T., Kuo Y.-H., Nguyen V.-B. (2021). Bioprocessing of Marine Chitinous Wastes for the Production of Bioactive Prodigiosin. Molecules.

[64] Hegazy A.A., Abu-Hussien S.H., Elsenosy N.K., El-Sayed S.M., Abo El-Naga M.Y. (2024). Optimization, Characterization and Biosafety of Carotenoids Produced from Whey Using Micrococcus Luteus. BMC Biotechnol..

[65] Gholami M., Etemadifar Z., Bouzari M. (2015). Isolation a New Strain of *Kocuria rosea* Capable of Tolerating Extreme Conditions. J. Environ. Radioact..

[66] Nemer G., Louka N., Vorobiev E., Salameh D., Nicaud J.-M., Maroun R.G., Koubaa M. (2021). Mechanical Cell Disruption Technologies for the Extraction of Dyes and Pigments from Microorganisms: A Review. Fermentation.

[67] Vachali P., Bhosale P., Bernstein P.S. (2012). Microbial Carotenoids. Microbial Carotenoids From Fungi: Methods in Molecular Biology.

[68] Volk W.A., Pennington S. (1950). The pigments of the photosynthetic bacterium *Rhodomicrobium vannielii*. J. Bacteriol..

[69] Chi S.C., Mothersole D.J., Dilbeck P., Niedzwiedzki D.M., Zhang H., Qian P., Vasilev C., Grayson K.J., Jackson P.J., Martin E.C. (2015). Assembly of Functional Photosystem Complexes in Rhodobacter Sphaeroides Incorporating Carotenoids from the Spirilloxanthin Pathway. Biochim. Biophys. Acta (BBA)-Bioenerg..

[70] Bóna-Lovász J., Bóna A., Ederer M., Sawodny O., Ghosh R. (2013). A Rapid Method for the Extraction and Analysis of Carotenoids and Other Hydrophobic Substances Suitable for Systems Biology Studies with Photosynthetic Bacteria. Metabolites.

[71] Autenrieth C., Ghosh R. (2019). The Methoxylated, Highly Conjugated C40 Carotenoids, Spirilloxanthin and Anhydrorhodovibrin, Can Be Separated Using High Performance Liquid Chromatography with Safe and Environmentally Friendly Solvents. Metabolites.

[72] Izquierdo-Barba I., García-Martín J.M., Álvarez R., Palmero A., Esteban J., Pérez-Jorge C., Arcos D., Vallet-Regí M. (2015). Nanocolumnar Coatings with Selective Behavior towards Osteoblast and Staphylococcus Aureus Proliferation. Acta Biomater..

[73] Shanmuga Leela A., Jaya Lakshmi S.S., Leela K.V., Tanuj M.L., George M.G., Jayaprakash V. (2024). *Kocuria rosea* Sepsis in an Immunocompromised Patient: A Case Report. Cureus.

[74] Guardado-Fierros B.G., Lorenzo-Santiago M.A., Gumiere T., Aid L., Rodriguez-Campos J., Contreras-Ramos S.M. (2025). Glyphosate Biodegradation by Airborne Plant Growth-Promoting Bacteria: Influence on Soil Microbiome Dynamics. Agriculture.

[75] Es-haghi A., Amiri M.S., Taghavizadeh Yazdi M.E. (2024). Ferula Latisecta Gels for Synthesis of Zinc/Silver Binary Nanoparticles: Antibacterial Effects against Gram-Negative and Gram-Positive Bacteria and Physicochemical Characteristics. BMC Biotechnol..

[76] Li C.-J., Jiang Z.-M., Zhi X.-Y., Chen H.-H., Yu L.-Y., Li G.-F., Zhang Y.-Q. (2025). Genomic Insights into *Kocuria*: Taxonomic Revision and Identification of Five IAA-Producing Extremophiles. Front. Microbiol..

[77] Udensi J., Loskutova E., Loughman J., Byrne H.J. (2022). Quantitative Raman Analysis of Carotenoid Protein Complexes in Aqueous Solution. Molecules.

[78] Pallath N., Francis B., Devanesan S., Farhat K., Balakrishnan M. (2023). Isolation and Characterization of Novel Carotenoid Pigment from Marine *Planococcus maritimus* MBP-2 and Their Biological Applications. J. King Saud Univ. Sci..

[79] Lestari S.W., Hertika A.M.S., Yona D., Buwono N.R. (2025). Functional Groups in Microalgal Extracellular Polymeric Substances: A Promising Biopolymer for Microplastic Mitigation in Marine Ecosystems. Ecol. Eng. Environ. Technol..

[80] Karacaoğlu B., Koçer A.T., İnan B., Bütün İ., Mercimek R., Ghorbani M., Koşar A., Balkanlı D. (2025). Microfluidic Chip-Assisted Separation Process and Post-Chip Microalgae Cultivation for Carotenoid Production. J. Appl. Phycol..

[81] Sharma R., Singh J., Verma N. (2018). Production, Characterization and Environmental Applications of Biosurfactants from *Bacillus amyloliquefaciens* and *Bacillus subtilis*. Biocatal. Agric. Biotechnol..

[82] Hendawy S.H.M., Alzan H.F., Abdel-Ghany H.S.M., Suarez C.E., Kamel G. (2024). Biochemical Analysis of *Hyalomma dromedarii* Salivary Glands and Gut Tissues Using SR-FTIR Micro-Spectroscopy. Sci. Rep..

[83] Kassem A., Abbas L., Coutinho O., Opara S., Najaf H., Kasperek D., Pokhrel K., Li X., Tiquia-Arashiro S. (2023). Applications of Fourier Transform-Infrared Spectroscopy in Microbial Cell Biology and Environmental Microbiology: Advances, Challenges, and Future Perspectives. Front. Microbiol..

